# Wasserstein Distance Learns Domain Invariant Feature Representations for Drift Compensation of E-Nose

**DOI:** 10.3390/s19173703

**Published:** 2019-08-26

**Authors:** Yang Tao, Chunyan Li, Zhifang Liang, Haocheng Yang, Juan Xu

**Affiliations:** School of Communication and Information Engineering, Chongqing University of Posts and Telecommunications, Chongqing 400065, China

**Keywords:** drift compensation, domain adaption, feature representations, electronic nose

## Abstract

Electronic nose (E-nose), a kind of instrument which combines with the gas sensor and the corresponding pattern recognition algorithm, is used to detect the type and concentration of gases. However, the sensor drift will occur in realistic application scenario of E-nose, which makes a variation of data distribution in feature space and causes a decrease in prediction accuracy. Therefore, studies on the drift compensation algorithms are receiving increasing attention in the field of the E-nose. In this paper, a novel method, namely Wasserstein Distance Learned Feature Representations (WDLFR), is put forward for drift compensation, which is based on the domain invariant feature representation learning. It regards a neural network as a domain discriminator to measure the empirical Wasserstein distance between the source domain (data without drift) and target domain (drift data). The WDLFR minimizes Wasserstein distance by optimizing the feature extractor in an adversarial manner. The Wasserstein distance for domain adaption has good gradient and generalization bound. Finally, the experiments are conducted on a real dataset of E-nose from the University of California, San Diego (UCSD). The experimental results demonstrate that the effectiveness of the proposed method outperforms all compared drift compensation methods, and the WDLFR succeeds in significantly reducing the sensor drift.

## 1. Introduction

Electronic nose (E-nose) is known as machine olfaction, consisting of the gas sensor array and corresponding pattern recognition algorithms, and is used to identify gases. Zhang et al. [1] and Wang et al. [2] used E-nose for air quality monitoring. Yan et al. [3] utilized E-nose to analysis disease. Rusinek et al. [4] used it for quality control of food. An increasing number of E-nose systems are being developed into actual applications because the E-nose systems are convenient to use, fast, and cheap. However, the sensor drift of E-nose still is a serious problem which decreases the performance of E-nose system and is receiving more and more attention. For most chemical sensors, sensor sensitivity may be influenced by many factors, such as environmental factors (temperature, humidity, pressure), self-aging and poisoning, etc. The change of sensor sensitivity will result in the fluctuation of sensor responses when the E-nose exposed to the same gas in different time, which is called the sensor drift [5]. In this paper, we mainly focus on the drift compensation of the sensor.

A number of methods have been applied to cope with the sensor drift of E-nose. From the perspective of signal preprocessing methods [6,7], frequency analysis and baseline manipulation have been adopted to compensate each sensor response. From the perspective of component correction, Artursson et al. [8] proposed the principal component analysis (PCA) method to correct the entire sensor response and suppress the sensor drift. The orthogonal signal correction (OSC) was proposed in [9,10] for the drift compensation of the E-nose. From the angle of the classifier, Vergara et al. [5]. proposed an ensemble strategy to enhance the robustness of the classifier and address the sensor drift. Dang et al. [11] proposed an improved support vector machine ensemble (ISVMEN) method, which improved classification accuracies and dealt with the sensor drift. In addition, some adaptive methods also are used to solve the problem of the sensor drift, such as the self-organizing map (SOM) [12], domain adaption methods [13], etc.

The above methods can suppress the drift to a certain extent, but the effects of these methods are limited due to the weak generalization of drift data. The sensor drift will make a variation of data distributions between the collected samples previously (data without drift) and the collected samples later (drift data). It will cause a great decrease in classification accuracies when the model trained with the data without drift is directly applied in testing samples with the drift. Therefore, in the sensor researches and pattern recognition communities, it is challenging to find a drift compensation algorithm with good robustness and adaptability.

In these cases, domain adaption techniques are a proper solution to deal with the problem of inconsistent data distributions between the source and the target domain samples. These techniques also have broad applications in many research fields, including natural language processing, machine vision, etc. [14,15,16]. In the drift compensation of the sensor, it is assumed that the data without drift is viewed as the source domain, and the drift data is considered as the target domain. At present, some scholars have performed domain adaption techniques on drift compensation algorithms. An intuitive idea is to reduce the difference of distributions among domains in the feature level, i.e., to learn domain-invariant feature representations. The geodesic flow kernel (GFK) method for the drift compensation was presented by Gong et al. [17], and it aimed to model the domain shift by integrating an infinite number of subspaces that describe the change in geometric and statistical properties from the source domain to the target domain. The advancement of the GFK for the drift compensation was presented in [18], namely domain adaption by shifting covariance (DASC). The mentioned methods have reduced the sensor drift to a certain extent. However, these domain adaption methods project the source and the target samples into a separate subspace, and the one domain, as a subspace, is not sufficient to represent the difference of distributions across domains. In this paper, we are committed to learn domain invariant feature representations in the common feature space, and some scholars performed made relevant researches. A domain regularization component analysis (DRCA) method was proposed by Zhang et al. [19] to map all samples from two domains to the same feature subspace, and it measured the distribution discrepancy between the source and the target domain using the maximum mean discrepancy (MMD). However, a linear mapping technique cannot seriously ensure “drift-less” properties in the E-nose. Ke Yan, Lu Kou et al. [20] minimized the distance between the source and target domain feature representations by maximizing the independence between data features and domain features (device label and acquisition time of a sample), which solved the issue of the sensor drift. A domain correction, based on the kernel transformation (DCKT) method, was proposed in [21]. It aligned the distributions of the two domains by mapping all samples to a high-dimensional reproducing kernel space and reduced the sensor drift. Some algorithms that have appeared in deep learning in recent years are applicable to guide feature representation learning. The representative features of the E-nose were extracted with autoencoders [22,23]. In addition, some adversarial domain adaption methods were also adopted to reduce the discrepancy across domains [24,25,26]. Arjovsky et al. [27] utilized Wasserstein distance to achieve a great breakthrough in the fields of common sentiments and images. However, there are few relative researches on Wasserstein distance to reduce the drift in E-nose.

Inspired by Wasserstein GAN (WGAN) [26] and spectral normalized GANs (SN-GANs) [27], a new drift compensation algorithm called Wasserstein Distance Learned Feature Representations (WDLFR) is proposed in this paper. First, the WDLFR measures the distribution discrepancy across domains using Wasserstein distance, and it estimates the empirical Wasserstein distance between feature representations of the source and the target domain by learning an optimal domain discriminator. Then, WDLFR minimizes the estimated empirical Wasserstein distance by constantly updating a feature extractor network in an adversarial manner. Finally, in order to make the extracted feature representations class-distinguished, the WDLFR incorporates the supervision information of the source domain samples into the feature representation learning. That is, the learned feature representations are domain-invariant and class-distinguished. Empirical studies on a dataset of E-nose from the University of California, San Diego (UCSD) demonstrate that the effectiveness of the proposed WDLFR outperforms the comparison approaches.

The rest of this paper is organized as follows. The basis of the proposed method is presented in Section 2. Section 3 details the proposed WDLFR approach based on the domain invariant feature representation learning. The experiments and results are discussed in Section 4. Finally, Section 5 concludes this paper. 

## 2. Related Work

In this section, a brief introduction of the Wasserstein distance will be given. It is the basis of the proposed method. 

### Wasserstein Distance

Wasserstein distance is used to measure the distance between two probability distributions and is defined as

(1) $$W[P,Q]=\underset{\gamma \in \prod [P,Q]}{\inf}\iint \gamma (x,y)\rho (x,y)dxdy$$

where ρ(x,y) is a cost function representing the cost of transportation from the instance *x* to *y*, and the common cost function is based on *l* norm, such as 1-norm ${\left\|x-y\right\|}_{1}$ and 2-norm ${\left\|x-y\right\|}_{2}$. Due to the equivalence property of norm, the final result of Wasserstein distance is closed. γ∈∏(P,Q) shows that γ is a joint distribution, satisfying constraint ∫γ(x,y)dy=P(x) and ∫γ(x,y)dx=Q(y) simultaneously, and P, Q are marginal distributions. In fact, Wasserstein distance metric appears in the problem of optimal transport: γ(x,y) is considered as a randomized scheme for transporting goods from a random location *x* to another random location *y*, and it satisfies the marginal constraint x∈P and x∈Q. If the cost of transporting a unit of goods from x∈P to x∈Q is given by ρ(x,y), W[P,Q] is the minimum expected transport cost.

The Kantorovich-Rubinstein theorem shows that the dual form of Wasserstein distance [28] can be written as follows

(2) $$W[P,Q]=\underset{{\left\|f\right\|}_{L}\leq 1}{\sup}E_{x∼P}\left[f\left(x\right)\right]-E_{x∼Q}\left[f\left(x\right)\right],$$

where the Lipschitz constraint is used to limit the change of function value and is defined as ${\left\|f\right\|}_{L}=\sup\left|f(x)-f(y)\right|/\rho (x,y)$. In this paper, for simplicity, Equation (2) is viewed as the final Wasserstein distance, and [29] has showed that Wasserstein distance has a good gradient and generalization bound for domain adaption under the Lipschitz constraint.

## 3. Wasserstein Distance Learned Feature Representations (WDLFR)

### 3.1. Problem Definition

In domain adaption techniques, the source and the target domain are defined by describing “S” and “T”, respectively. We have the training set $X_{S}={\left\{\left(x_{i}^{s},y_{i}^{s}\right)\right\}}_{i=1}^{n^{s}}$ of $n^{s}$ labeled samples from the source domain $D_{S}$, and the testing set is defined as $X_{t}={\left\{\left(x_{j}^{t}\right)\right\}}_{j=1}^{n^{t}}$ of $n^{t}$ unlabeled samples from the target domain $D_{T}$. It is assumed that the source and the target domain share the same feature space, but the marginal distributions are different ($P_{x^{s}}$ and $P_{x^{t}}$ respectively). The purpose of domain adaption is to reduce the divergence between the two domains, and the classifier of the source domain can be directly applied to the target domain.

### 3.2. Domain Invariant Feature Representation Learning

The sensor drift of the E-nose leads to inconsistent data distributions between the previously collected samples (source domain) and the later collected samples (target domain), which means that the model trained with source domain samples may be highly biased in the target domain. To solve this problem, a new method (WDLFR) is proposed in this paper. The learned feature representations are invariant to the change of domain by minimizing the empirical Wasserstein distance between the source and the target feature representations through an adversarial training manner. 

The adversarial method is composed of two parts, including the feature extractor and the domain discriminator implemented by a neural network. The feature extractor network is used to learn the feature representations of the source and the target domain, and domain discriminator is used to estimate the empirical Wasserstein distance between the feature representations of both domains. First, considering a sample of any domain $x\in R^{m}$, the feature extractor network learns a function $f_{g}:R^{m}\to R^{d}$ that maps the sample to a *d*-dimensional representation with the network parameters $\theta_{g}$. The feature representations can be calculated by $h=f_{g}\left(x\right)$, and the feature representation distributions of the source and the target domain are $P_{h^{s}}$ and $P_{h^{t}}$, respectively. Therefore, the Wasserstein distance between the feature representation distributions $P_{h^{s}}$ and $P_{h^{s}}$ can be expressed by Equation (2)

(3) $$W[P_{h^{s}},\;P_{h^{s}}]=\underset{{\left\|f\right\|}_{L}\leq 1}{\sup}E_{h∼P_{h^{s}}}\left[f\left(h\right)\right]-E_{h∼P_{h^{t}}}\left[f\left(h\right)\right].$$

For the function *f*, we can train a domain discriminator, as suggested in [28], to learn a function $f_{w}:R^{d}\to R$ that maps feature representations to a real number with corresponding network parameters $\theta_{w}$. The Wasserstein distance can be reformulated as

(4) $$W[P_{h^{s}},\;P_{h^{s}}]=\underset{{\left\|f\right\|}_{L}\leq 1}{\sup}E_{h∼P_{h^{s}}}\left[f_{w}\left(h\right)\right]-E_{h∼P_{h^{t}}}\left[f_{w}\left(h\right)\right].$$

According to the feature extractor network, the feature representations of the source and the target domain are $h^{s}=f_{g}\left(x^{s}\right)$ and $h^{t}=f_{g}\left(x^{t}\right)$, respectively. The Wasserstein distance between the feature representation distributions of the source and the target domain can, again, be written as follows

(5) $$\begin{array}{ll} W[P_{h^{s}},\;P_{h^{s}}] & =\underset{{\left\|f\right\|}_{L}\leq 1}{\sup}E_{h∼P_{h^{s}}}\left[f_{w}\left(h\right)\right]-E_{h∼P_{h^{t}}}\left[f_{w}\left(h\right)\right]\\ & =\underset{{\left\|f\right\|}_{L}\leq 1}{\sup}E_{x∼P_{x^{s}}}\left[f_{w}\left(f_{g}\left(x\right)\right)\right]-E_{x∼P_{x^{t}}}\left[f_{w}\left(f_{g}\left(x\right)\right)\right] \end{array}$$

If the function of the domain discriminator $f_{w}$ satisfies the Lipschitz constraint, and the Lipschitz norm is bounded to 1, the empirical Wasserstein distance can be approximated by maximizing the domain discriminator loss $l_{wd}$ with respect to parameters $\theta_{w}$

(6) $$\underset{\theta_{w}}{\max}\;l_{wd},$$

where the domain discriminator loss $l_{wd}$ is represented as

(7) $$l_{wd}(x^{s},x^{t})=\frac{1}{n^{s}}\sum_{x^{s}\in X_{s}}f_{w}(f_{g}(x^{s}))-\frac{1}{n^{t}}\sum_{x^{t}\in X_{t}}f_{w}(f_{g}(x^{t})).$$

Here, the question of enforcing the Lipschitz constraint is raised. A weight clipping method was presented in [26], aiming to limit all weight parameters of domain discriminator to the range of [−c,c] after each gradient update. However, [30] pointed out that it is easy to cause the problem of gradient disappearances and gradient explosions. Gulrajani et al. [30] proposed a more appropriate gradient penalty method to make the domain discriminator satisfy the Lipschitz constraint, and the method can obtain a good result in most cases. However, the linear gradient interpolation method can only ensure that the Lipschitz constraint is satisfied in a small space, and the interpolation between different label samples may not satisfy the Lipschitz constraint. The disadvantages are pointed out in [27]. As suggested in [27], a more reasonable method is to update the weight parameters $\theta_{w}$ by the spectral normalization method after each gradient update. The merit of the spectral normalization method is that the domain discriminator $f_{w}$ can satisfy the Lipschitz constraint no matter how the domain discriminator parameters $\theta_{w}$ change. Therefore, the spectral normalization method will be used to make the domain discriminator $f_{w}$ satisfy the Lipschitz constraint. 

Now, the Wasserstein distance is continuous and differentiable almost everywhere, and an optimal domain discriminator can be trained first. Then, by fixing the optimal network parameters of domain discriminator and minimizing the Wasserstein distance, the feature extractor network can learn the feature representations with the domain discrepancy reduced. Therefore, the feature representations can be estimated by solving the minimax problem

(8) $$\underset{\theta_{g}}{\min}\;\underset{\theta_{w}}{\max}\;l_{wd}$$

Finally, by iteratively learning feature representations with lower Wasserstein distance, the adversarial objective function can learn domain invariant feature representations.

### 3.3. Combing with Supervision Signals

**Figure 1:**
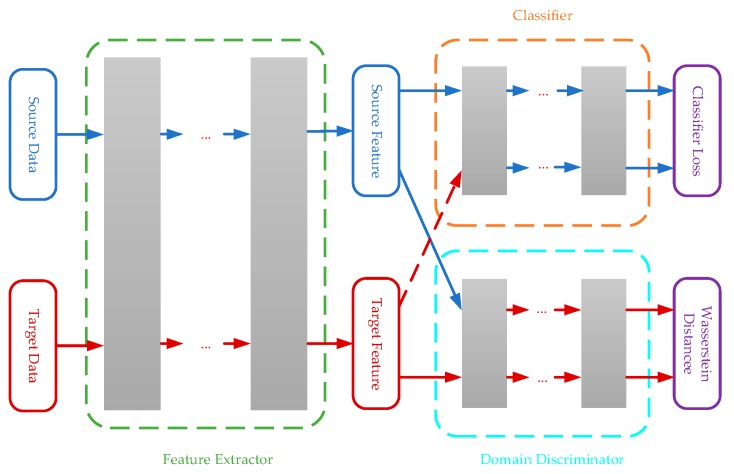
Wasserstein Distance Learned Feature Representations (WDLFR) combined with the classifier.

The final purpose of this paper is to ensure that the classifier of the source domain will have a good performance in the target domain. Considering the above domain adaption method, it may be impossible to learn the optimal feature representations. Because the WDLRF method can learn domain invariant feature representations and guarantees transferability of the learned feature representations, the source domain classifier is feasible to the target domain. However, the learned domain invariant feature representations are not enough class-distinguished. Therefore, the supervision information of the training set denoted by the source domain will be integrated into the domain invariant feature representation learning as suggested in [24]. The overview framework of the algorithm is shown in Figure 1.

Next, the combination of the feature representation learning and the classifier will be introduced. Several layers can be added as the classifier after the feature extractor network. The objective of the classifier $f_{c}:R^{d}\to R^{l}$ is to compute the Softmax prediction with the network parameters $\theta_{c}$, where l is the number of classes. The Softmax prediction is mainly used in multi-classification problems, and it will divide the entire space according to the number of classes to ensure that the classes are separable. Finally, the empirical loss of the classifier in the source domain is given by

(9) $$l_{c}(x^{s},y^{s})=\underset{\theta_{c}}{\min}\sum_{i=1}^{n^{s}}L(f_{c}(x_{i}^{s}),y_{i}^{s}),$$

where $L\left(f_{c}(x_{i}^{s}),y_{i}^{s}\right)$ is the cross-entropy between the predicted probabilistic distribution and the one-hot encoding of the class labels given the labeled source data

(10) $$L(f_{c}(x_{i}^{s}),y_{i}^{s})=-\sum_{k=1}^{l}1(y_{i}^{s}=k)\cdot \log f_{c}{\left(f_{g}(x_{i}^{s})\right)}_{k}.$$

$1\left(y_{i}^{s}=k\right)$ is an indicator function, and $\log f_{c}{\left(f_{g}(x_{i}^{s})\right)}_{k}$ corresponds to the *k*-th dimension value of the distribution $f_{c}(f_{g}(x_{i}^{s}))$. Therefore, the final empirical loss of the source domain classifier is expressed as

(11) $$l_{c}(x^{s},y^{s})=\underset{\theta_{c}}{\min}-\frac{1}{n^{s}}\sum_{i=1}^{n^{s}}\sum_{k=1}^{l}1(y_{i}^{s}=k)\cdot \log f_{c}{\left(f_{g}(x_{i}^{s})\right)}_{k}.$$

Finally, the final objective function is obtained by combining the Equations (8) with (11)

(12) $$\underset{\theta_{g},\theta_{c}}{\min}(l_{c}+\lambda \underset{\theta_{w}}{\max l_{wd}}),$$

where λ is a coefficient parameter used to control the balance between class-distinguished and transferable feature representation learning. The process of the WDLFR is shown in Algorithm 1.

The WDLFR algorithm can be implemented using the standard back-propagation with two iterations. In a mini-batch size including labeled source data and unlabeled target data, the domain discriminator can firstly be trained to optimal point by gradient ascent. The mini-batch gradient ascent method divides all samples of two domains into several batches and updates the network parameters of the neural network in each batch way, which can reduce the computational complexity. In other words, the mini-batch gradient ascent method divides the training set into several small training sets. Then, in order to reduce the distribution discrepancy across domains, we simultaneously minimize the estimated empirical Wasserstein distance across domains and the classification loss computed by labeled source samples to update the feature extractor network. Finally, the learned feature representations are domain-invariant and class-distinguished, since the parameter $\theta_{g}$ receives the gradients from both the domain discriminator and the classifier. 

The sensor drift changes the features of the collected data. Furthermore, it also makes data distributions different. Domain adaption techniques can reduce the difference of distributions among domains. Therefore, the proposed WDLRF method can be used to reduce the drift of the E-nose. 

**Algorithm 1** The Proposed WDLFR Method: Asserstein Distance Learned Feature Representations Combining with Classifier**Require:** Labeled source data $X_{S}$, unlabeled target data $X_{t}$, mini-batch size m, total training iterations n, training step of domain discrimination k, coefficient parameter λ, learning rate of domain discrimination α, learning rate of features representations learning and classifier β.1. Initialize feature extractor, domain discrimination, classifier with random weights $\theta_{g}$, $\theta_{w}$, $\theta_{c}$
2. **Repeat:** (total training iterations n)3.  Sample m instances ${\left\{\left(x_{i}^{s},y_{i}^{s}\right)\right\}}_{i=1}^{m}$ from $X_{S}$
  Sample m examples ${\left\{\left(x_{i}^{t}\right)\right\}}_{i=1}^{m}$ from $X_{t}$
4.  **For**
*i* = 1, …, *k*
**do**5.   $h^{s}\leftarrow f_{g}(x^{s})$, $h^{t}\leftarrow f_{g}(x^{t})$
6.   $\theta_{w}\leftarrow \theta_{w}+\alpha \nabla_{\theta_{w}}[l_{wd}(x^{s},x^{t})]$
7.   Calculate spectral normalization weights ${\bar{W}}_{SN}$
8.   $\theta_{w}\leftarrow \theta_{w}+\alpha \nabla_{\theta_{w}}[l_{wd}(x^{s},x^{t})]$
9. **End for**10. $\theta_{c}\leftarrow \theta_{c}-\beta \nabla_{\theta_{c}}[l_{c}(x^{s},y^{s})]$
11. $\theta_{g}\leftarrow \theta_{g}-\beta \nabla_{\theta_{g}}[l_{c}(x^{s},y^{s})+l_{wd}(x^{s},x^{t})]$
12. **Until**
$\theta_{g}$, $\theta_{w}$, $\theta_{c}$ converge

## 4. Experiments

In this section, the real sensor drift benchmark dataset of the E-nose from UCSD is used to evaluate the effectiveness of the WDLFR method, and the experimental results of the proposed WDLFR method are compared with that of other drift compensation algorithms in E-nose.

### 4.1. Sensor Drift Benchmark Dataset

The real sensor drift benchmark dataset, consisting of data from three years, was collected by Vergara et al. [5] at UCSD. The sensor array of the E-nose was composed of 16 chemical sensors, each of which extracted 8 features of the sample. Consequently, each sample had a total of 128 (16 × 8) dimensional feature vectors. The E-nose was utilized to measure six gases (acetone, acetaldehyde, ethanol, ethylene, ammonia, and toluene) at different concentrations. A total of 13,910 samples were gathered over a course of 36 months from January 2008 to February 2011, which were split into 10 batches according to the chronological order. The sensor response information of the tenth batch was collected deliberately after the E-nose was powered off for five months. These sensors were susceptible to serious pollution during the five months, and the pollution was irreversible so that the operating temperature of the chemical sensor array in the sensor chamber was not able to resume to a normal extent. In this situation, serious drift will happen on the collected samples. Therefore, the tenth batch will suffer from the most serious drift compared to other batches. The period of collection and the number of samples for each class, with respect to each batch, are summarized in Table 1. More information on the real sensor drift benchmark dataset can be found in [5]. 

**Figure 2:**
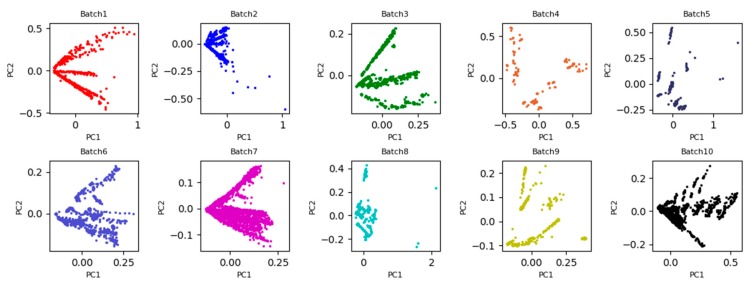
Two-dimensional principle component (PC1, PC2) scatter points of 10 batches data by principal component analysis (PCA).

To more intuitively observe the data distribution discrepancy of all batches, the two-dimensional principle component scatter points of the original data are plotted in Figure 2. From Figure 2, it is clearly observed that the 2D subspace distribution between Batch 1 and the other batches is significantly inconsistent due to the impact of the sensor drift. If Batch 1 is considered as the source domain for training model, test on Batch b, b = 2, …, 10 (i.e., target domain), the recognition accuracy will create a great bias. One possible reason is that it violates the basic assumptions of machine learning: The training set and the test set should maintain the same or similar probability distribution. In this case, the distributions between the two domains can be aligned by learning the domain invariant feature representations.

### 4.2. WDLRF Implementation Details

In this paper, all experiments are performed using Tensorflow and the training model is optimized using Adam optimizer. The advantage of the Adam optimizer is that each iteration of the learning rate has a clear range, and it makes the change of parameters very stable. Under the situation of the best experimental results, the constructions of the WDLRF method are as follows. The feature extractor network contains an input layer of 128 neuron nodes and an output layer of 200 neuron nodes. The domain discriminator is designed with an input layer of 200 nodes, one hidden layer of 10 nodes, and an output layer of 1 node. The classifier is composed with an input layer of 200 nodes and an output layer of 6 nodes. All the activation functions adopt the ReLU function, except a Softmax function for the classifier. After normalizing all samples from source and target domains, the samples from the source and target domain are first inputted into the feature extractor network. Then, the extracted source domain features are inputted into the classifier, and the source and target domain features are inputted into the domain discriminator to evaluate the Wasserstein distance. Finally, it updates the feature extractor network by learning the classifier and the domain discriminator at the same time. Therefore, the extracted features from the feature extractor network have domain invariant characteristics, and the distribution consistencies of source and target domain are greatly improved.

### 4.3. The Experiment Results and Analysis

The classification accuracies are used as a criterion to judge the drift reduction. A goal that the WDLFR method aligns with the distribution of the source and target domain is to improve the performance of the classifier. Therefore, all experiments are conducted under Experimental Setting 1 and Setting 2. In order to better verify the effectiveness of the proposed WDLFR method in this paper, the proposed approach compares with principal component analysis (PCA) [8], linear discriminant analysis (LDA) [31], domain regularized component analysis (DRCA) [19], and SVM ensemble (SVM-rbf, SVM-comgfk).

Setting 1: Take Batch 1 as the source domain for training model, and test on Batch b, b = 2, 3, …, 10.

Setting 2: Take Batch b as the source domain for training model, and test on Batch (b + 1), b = 1, 2, 3, …, 9.

**Figure 3:**
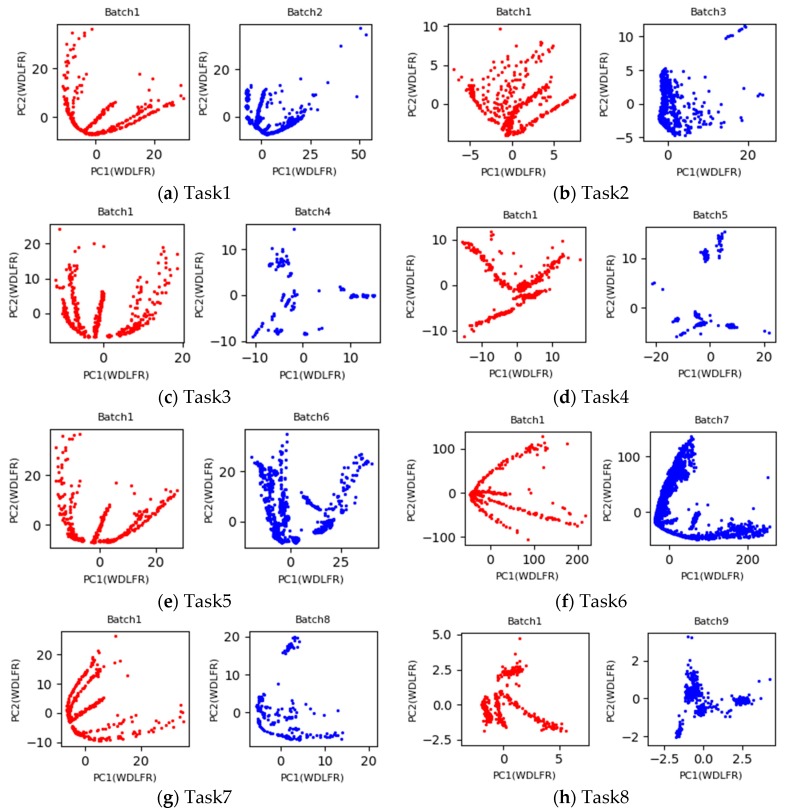

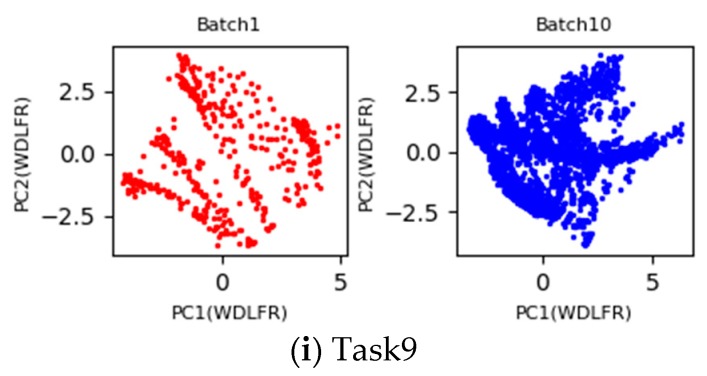
Two-dimensional principle component scatter points of the source and target domain feature representations after using the proposed WDLFR method.

Under the setting 1, the first batch with labeled data is used as the source domain, and the b-th (b = 2, 3, …, 10) batch with unlabeled data is considered as the target domain. If the process of learning domain invariant feature representations from the both domains is regarded as a task, a total of nine tasks with pair-wise batches (Batch 1 vs. Batch b) (b = 2, 3, …, 10) are implemented in this Experimental Setting 1. Inspired by PCA scatter points in Figure 2, the 2D principle component scatter points of nine tasks after using the proposed WDLFR method are shown in Figure 3. Comparing the PCA scatter points in Figure 2 and Figure 3, the distribution discrepancy between the source and target data has been greatly reduced in a certain extent, and the distribution consistencies have been improved greatly. Therefore, the classifier trained with source data is feasible to target data.

**Figure 4:**
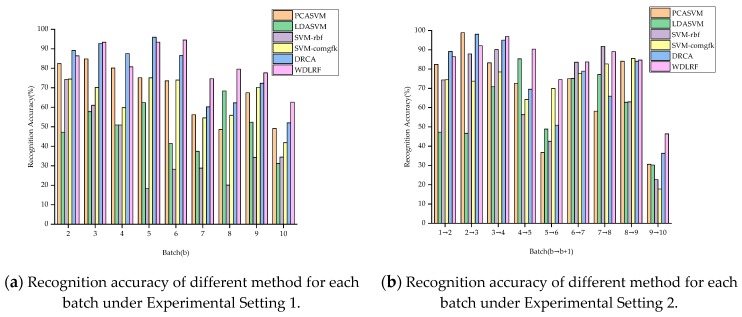
Recognition accuracy bar chart under Experimental Setting 1 and Setting 2.

In order to represent the effect of the proposed WDLFR method on drift suppression intuitively, the recognition results of all comparison algorithms under Experimental Setting 1 are reported in Table 2. First, it can be found that the average recognition accuracy of the WDLFR is the best, reaching 82.55%. The average recognition accuracy is 4.92% higher than the second-best performance method (i.e., DRCA). Second, the recognition accuracy of Batch 10 is the lowest, rather than other batches. One possible reason is that the data of Batch 10 are gathered after the E-nose is powered off for five months, which will cause the data of Batch 10 to experience the more serious drift. Therefore, it is difficult to align the marginal probability between Batch 10 and Batch 1. Overall, the proposed WDLFR method is feasible for drift compensation. In addition, in order to reflect the effectiveness of each method intuitively, the recognition accuracy bar chart of each method under Experimental Setting 1 is drawn in Figure 4a. From Figure 4a, it can be clearly seen that the recognition accuracy for the most of batches is much higher than other compared approaches. Since the proposed WDLFR method adopts the mini-batch gradient ascent training way, the mini-batch size setting under the highest accuracy of each task is given in Table 3.

Under Experimental Setting 2, the b-th batch of dataset is used as source data, and the (b + 1)-th batch of dataset is viewed as target data, b = 1, 2, 3, …, 9, i.e., the classification model is trained on Batch b and tests on Batch (b + 1). The experimental comparison results of recognition accuracy for each algorithm are reported in Table 4, and the corresponding parameters (mini-batch size) are shown in Table 5. From Table 4, it can be clearly observed that the proposed algorithm achieves the highest average recognition accuracy, reaching 83.08%. The average recognition accuracy is 8.86% higher than the second-best performance method (i.e., DRCA). The recognition accuracy bar chart of each method is drawn in Figure 4b. From Figure 4b, the WDLFR attains the highest performance for the most of batches. Overall, the experimental comparison results under Setting 1 and Setting 2 confirm the fact that the proposed WDLFR method has a great advancement for reduction drift. At the same time, the effectiveness and competitiveness of the proposed WDLFR method are demonstrated.

## 5. Conclusions

In order to solve the issue of inconsistent distributions caused by the sensor drift in the E-nose, a novel drift compensation algorithm (WDLFR) is proposed in this paper. The WDLFR can effectively reduce the distribution discrepancy by taking the merit of the good gradient property and generalization bound of Wasserstein distance. Furthermore, the WDLFR can reduce the drift of the E-nose. The characteristics of the WDLFR are as follows: (1) The feature extractor network and the domain discriminator are trained by an adversarial manner, so that the extracted features by the feature extractor network can eventually cheat the domain discriminator to generate domain invariant feature representations; (2) It combines the feature extractor with classifier to make the learned domain invariant feature representations class-distinguished. Finally, in order to verify the effectiveness of the WDLFR, we experiment on the dataset of the E-nose from USCD, and the classification accuracy is better than other comparison algorithms using the proposed WDLFR method.

In the future, we will continue to expand the work from the perspective of adaption classifier. It establishes a residual relationship between the source and the target domain classifier, and combines the feature extractor network with the adaption classifier to compensate the drift.

## Figures and Tables

**Table 1:** Sensor drift benchmark dataset.

Batch ID	Month	Acetone	Acetaldehyde	Ethanol	Ethylene	Ammonia	Toluene
Batch 1	1, 2	90	98	83	30	70	74
Batch 2	3~10	164	334	100	109	532	5
Batch 3	11~13	365	490	216	240	275	0
Batch 4	14, 15	64	43	12	30	12	0
Batch 5	16	28	40	20	46	63	0
Batch 6	17~20	514	574	110	29	606	467
Batch 7	21	649	662	360	744	630	568
Batch 8	22, 23	30	30	40	33	143	18
Batch 9	24, 30	61	55	100	75	78	101
Batch 10	36	600	600	600	600	600	600

**Table 2:** Recognition Accuracy (%) under Experimental Setting 1. The bold font represents the highest recognition accuracy of a batch in all compared algorithms.

Methods	Batch ID									Average
	2	3	4	5	6	7	8	9	10	
PCA_SVM_	82.40	84.80	80.12	75.13	73.57	56.16	48.64	67.45	49.14	68.60
LDA_SVM_	47.27	57.76	50.93	62.44	41.48	37.42	68.3**7**	52.34	31.17	49.91
SVM-rbf	74.36	61.03	50.93	18.27	28.26	28.81	20.07	34.26	34.47	38.94
SVM-comgfk	74.47	70.15	59.78	75.09	73.99	54.59	55.88	70.23	41.85	64.00
DRCA	**89.15**	92.69	**87.58**	**95.94**	86.52	60.25	62.24	72.34	52.00	77.63
WDLRF	86.41	**93.38**	80.75	93.40	**94.48**	**74.65**	**79.59**	**77.66**	**62.64**	**82.55**

**Table 3:** Corresponding Parameter Setting (mini-batch size) of the WDLFR under Experimental Setting 1.

BatchID	2	3	4	5	6	7	8	9	10
Mini-batch size	12	12	32	16	32	64	14	16	16

**Table 4:** Recognition Accuracy (%) under Experimental Setting 2. The bold font represents the highest recognition accuracy of a batch in all compared algorithms.

Methods	Batch ID									Average
	1 → 2	2 → 3	3 → 4	4 → 5	5 → 6	6 → 7	7 → 8	8 → 9	9 → 10	
PCA**_SVM_**	82.40	**98.87**	83.23	72.59	36.70	74.98	58.16	84.04	30.61	69.06
LDA_SVM_	47.27	46.72	70.81	85.28	48.87	75.15	77.21	62.77	30.25	60.48
SVM-rbf	74.36	87.83	90.06	56.35	42.52	83.53	**91.84**	62.98	22.64	68.01
SVM-comgfk	74.47	73.75	78.51	64.26	69.97	77.69	82.69	85.53	17.76	69.40
DRCA	89.15	98.11	95.03	69.54	50.87	78.94	65.99	84.04	36.31	74.22
WDLFR	86.41	92.13	**9** **6.89**	**9** **0.36**	**7** **4.57**	**83.70**	89.12	**84.68**	**46.42**	**83.** **08**

**Table 5:** Corresponding Parameter Setting (mini-batch size) of the WDLFR under Experimental Setting 2.

Batch ID	1 → 2	2 → 3	3 → 4	4 → 5	5 → 6	6 → 7	7 → 8	8 → 9	9 → 10
Mini-batch size	12	16	32	32	12	64	14	12	16
